# Nonsmooth Convex Optimization for Structured Illumination Microscopy Image Reconstruction

**DOI:** 10.1088/1361-6420/aaccca

**Published:** 2018-07-12

**Authors:** Jérôme Boulanger, Nelly Pustelnik, Laurent Condat, Lucie Sengmanivong, Tristan Piolot

**Affiliations:** 1 CNRS UMR144, F-75248 Paris, France; 2 Institut Curie, F-75248 Paris, France; 3 Cell Biology Division, MRC Laboratory of Molecular Biology, Cambridge CB2 0QH, UK; 4 Laboratoire de Physique ENS de Lyon; 5 CNRS UMR5672, Université Lyon I, France; 6 CNRS, GIPSA-Lab, Univ. Grenoble Alpes, 38000 Grenoble, France; 7 Cell and Tissue Imaging Core Facility (PICT-IBiSA), F-75248 Paris, France; 8 Nikon Imaging Centre@Institut Curie-CNRS, F-75248 Paris, France; 9 CNRS UMR3215, F-75248, Paris, France; 10 INSERM U934, F-75248, Paris, France

## Abstract

In this paper, we propose a new approach for structured illumination microscopy image reconstruction. We first introduce the principles of this imaging modality and describe the forward model. We then propose the minimization of nonsmooth convex objective functions for the recovery of the unknown image. In this context, we investigate two data-fitting terms for Poisson-Gaussian noise and introduce a new patch-based regularization method. This approach is tested against other regularization approaches on a realistic benchmark. Finally, we perform some test experiments on images acquired on two different microscopes.

## 1 Introduction

### Context

**Figure 1:**
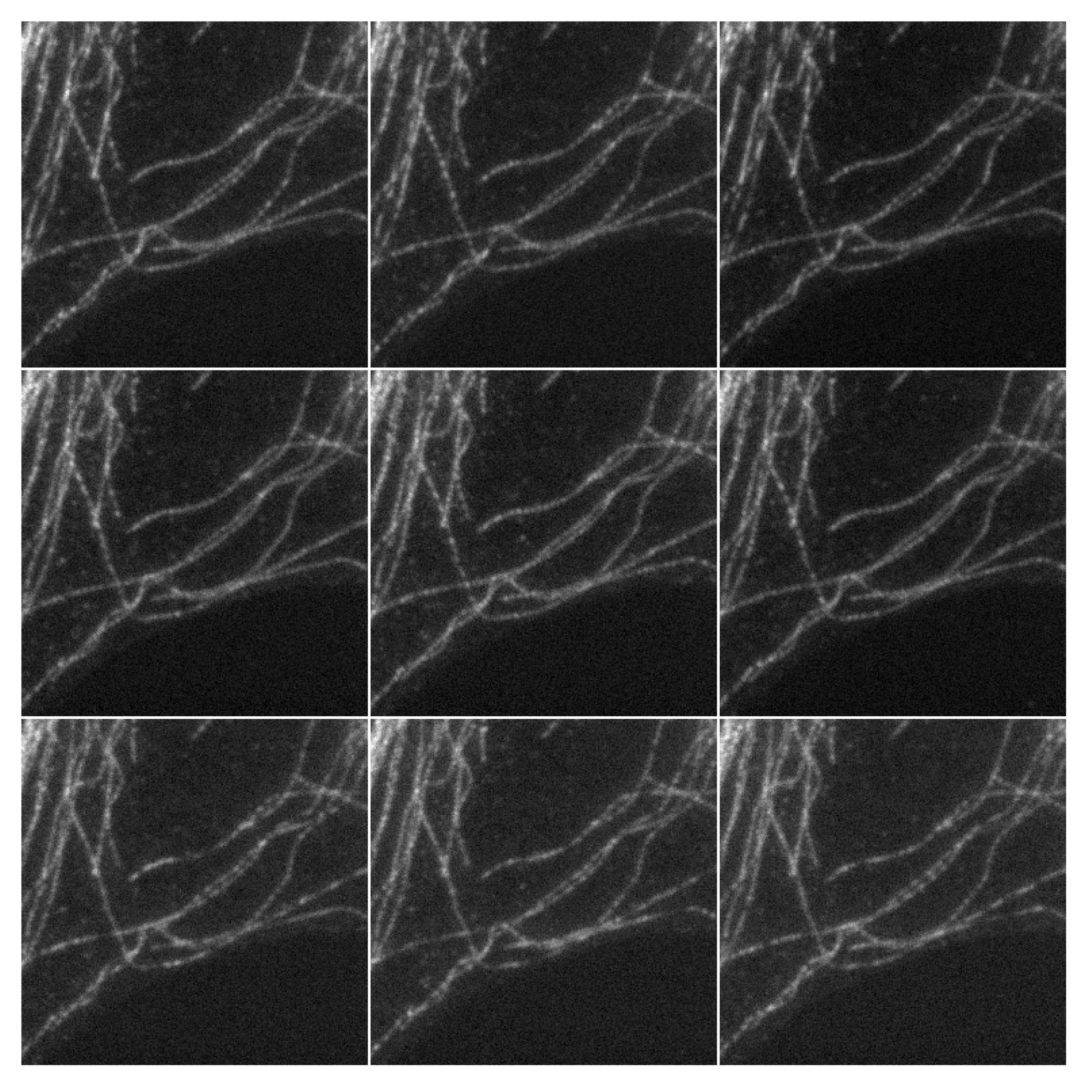
Example of real data. A Molecular Probe slide was imaged 9 times using a Nikon SIM microscope using a 100× oil objective. The images represent a 256 × 256 region of 512 × 512 acquired images and display some labeled microtubules. The modulation pattern can be observed as a slight Moiré effect on the object.

**Figure 2:**
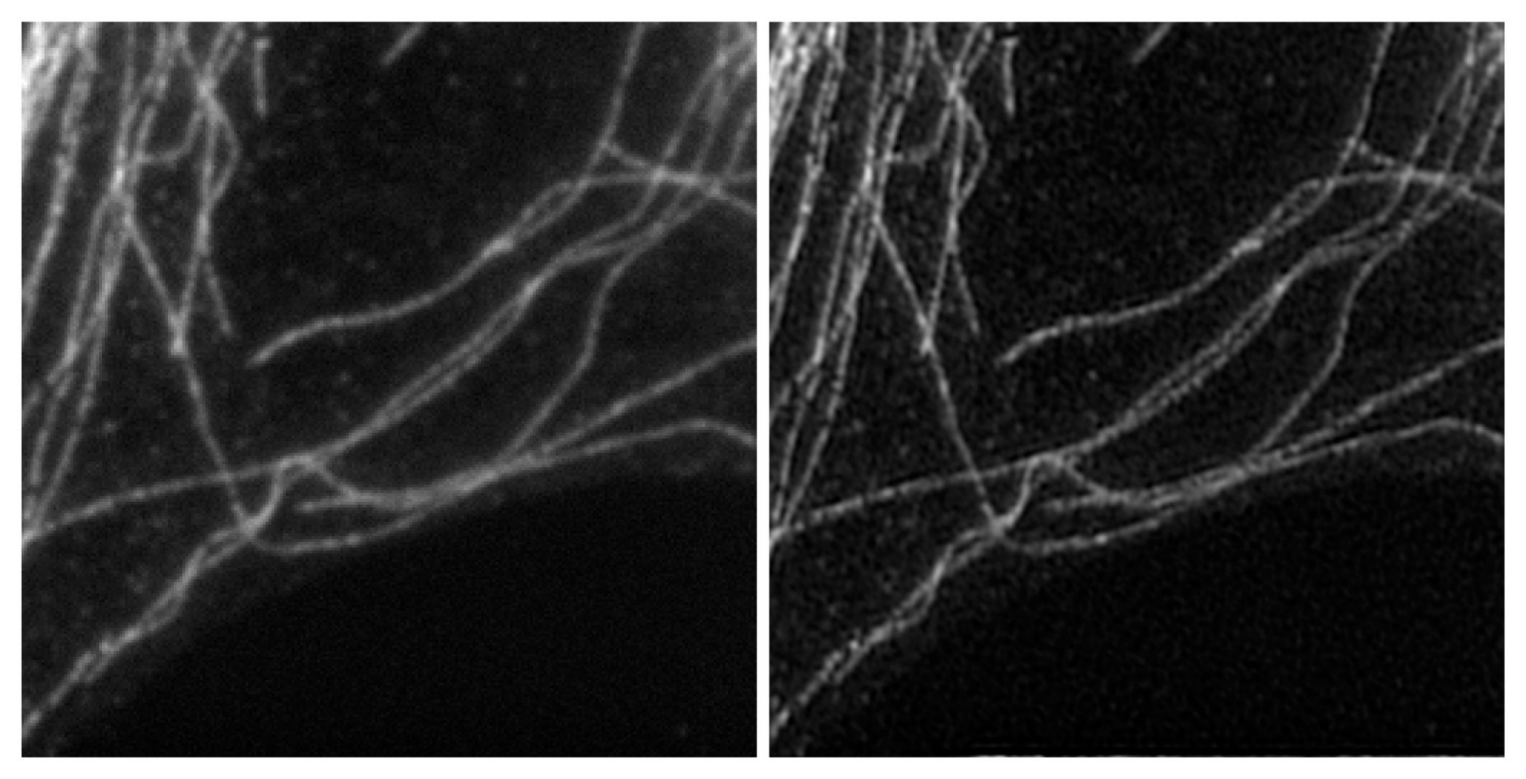
Reconstruction of the data displayed in Fig. 1. On the left the corresponding classical wide–field microscopy is obtained from the mean of the nine images. On the right, a linear least-squares reconstruction. The actual dimension of the image on the right is twice the size of the image of the left.

Superresolution approaches allow us to go beyond the resolution of standard widefield fluorescence microscopy, therefore breaking the classical diffraction limit defined by Abbe in 1873 [1]. Structured illumination microscopy (SIM) is one of the recently proposed optical superresolution methods compatible with time lapse imaging of several labels. Based on the illumination of a sample by a set of interference patterns, this technique makes it possible to typically increase the resolution of the microscope by a factor of two [2,3]. The resulting sinusoidal modulations of the fluorophore excitation signal lead to frequency shifts in the Fourier domain, which bring inaccessible frequencies within the scope of the optical transfer function of the microscope. An example of acquired raw data is depicted in Fig. 1. Once post-processed, the acquired images show an increased resolution, as illustrated in Fig. 2, where an acquired image has been reconstructed using a linear method [3]. Several studies have investigated the properties of such reconstruction algorithms and provided solutions for artifact reduction [4, 5]. However, like in many optical microscopy approaches, the photon counting process leads to noisy data, compromising the quality and the resolution of the final images. Therefore, the development of reconstruction methods less sensitive to noise and able to deal with the specificity of the structure of the reconstruction problem is crucially needed.

### Related work

While Wiener filtering remains the main reconstruction approach for SIM, the problem was recast in [6] as a more general inverse problem, allowing more complex illumination pattern to be considered [7–9]. Several regularization approaches have been also explored, in [6, 10] the *£*_2_ norm of the Laplacian operator is considered and total-variation (TV) was explored in [11] to deal with low signal to noise ratios. If [12] makes the underlying assumption of the Poisson noise model, none of these approaches consider a more accurate Poisson–Gaussian noise model. Moreover, none of the recent regularization methods, such as the Schatten norm of the Hessian operator [13], nonlocal total variation (NLTV) [14–16], global patch dictionaries [17, 18] or local patch dictionaries [19] have been applied to structured illumination reconstruction, therefore limiting the final performance of this superresolution technique in its ability to discriminate fine structures of interest.

### Contribution & organization of the article

We propose here a reconstruction method taking into account the Poisson–Gaussian distribution of the noise and relying on a new regularization approach based on learning local dictionaries of patches in a convex setting. The minimization of the resulting cost function is performed using a versatile primal–dual optimization method. An extensive comparison with alternative regularization approaches is provided and we detail the implementation aspects of the tested regularization approaches in an unified way. Note that this work is an extension of the method published in a conference proceedings [20].

We will first recall the image formation problem in Section. 2.2, and further introduce the proposed reconstruction scheme based on Poisson-Gaussian approximation and local dictionaries of patches in Section 3. A performance evaluation on a synthetic dataset is then detailed in Section 4.2 and the reconstruction of acquired data is finally analyzed in Section 4.4. Finally, In Appendix A, we recall the implementation details for the tested regularization cost terms and the needed tools for their minimization.

## 2 Presentation of the problem

### 2.1 Notations

In this article, **I**_*N*_ denotes the identity operator/matrix of size *N* × *N*; when the size is not mentioned, it should be clear from the context. · * denotes the adjoint of an operator; when the operator is assimilated to its representation matrix, with real entries, · * = · ^T^, the transpose operation. In the following, ⊗ denotes the Kronecker product and · ^†^, the Moore–Penrose pseudo-inverse. Notations used in this article are listed in Table 6.

### 2.2 Forward problem

Let us consider a set of *K* noise-free images **ȳ**_*k*_ with *k* = 1, … , *K*:

(1) $${\bar{\text{y}}}_{k}={\text{S}}_{0}{\text{A}}_{0}{\text{M}}_{k}\bar{\text{x}},$$

where **x̄** is the unknown two–dimensional image defined on a regular grid of size *N*_1_ × *N*_2_ and represented in a vectorized form by a vector of size *N* = *N*_1_*N*_2_. **M**_*k*_, **A**_0_ and **S**_0_ are three linear operators represented by matrix multiplications and corresponding to modulation, convolution and down-sampling, respectively.

The modulation operator **M**_*k*_ performs a pixelwise multiplication by a pattern image **m**_*k*_, so that **M**_*k*_ = diag(**m**_*k*_). In structured illumination microscopy, modulations often result from the interference of two or three coherent laser beams [2, 21] and can be represented by a sinusoidal pattern defined for each point of coordinates (*n*_1_, *n*_2_) ∈ *N*_1_ × *N*_2_ as:

(2) $${\left[{\text{m}}_{k}\right]}_{n_{1},n_{2}}=1+\alpha_{k}\text{cos}(n_{1}\omega_{1,k}+n_{2}\omega_{2,k}+\varphi_{k}),$$

where *α_k_* is the amplitude of the modulation, *ω*_1,*k*_ and *ω*_2,*k*_ are the modulation frequencies and *φ_k_* a phase. However, one can devise other light structuring strategies such as a set of scanning points [22], or random illumination [7], often at the expense of the number of required images. In the following, we will stack all the modulations **M**_*k*_ in the matrix **M** = [**M**_1_, … , **M**_*K*_]^T^.

**Figure 3:**
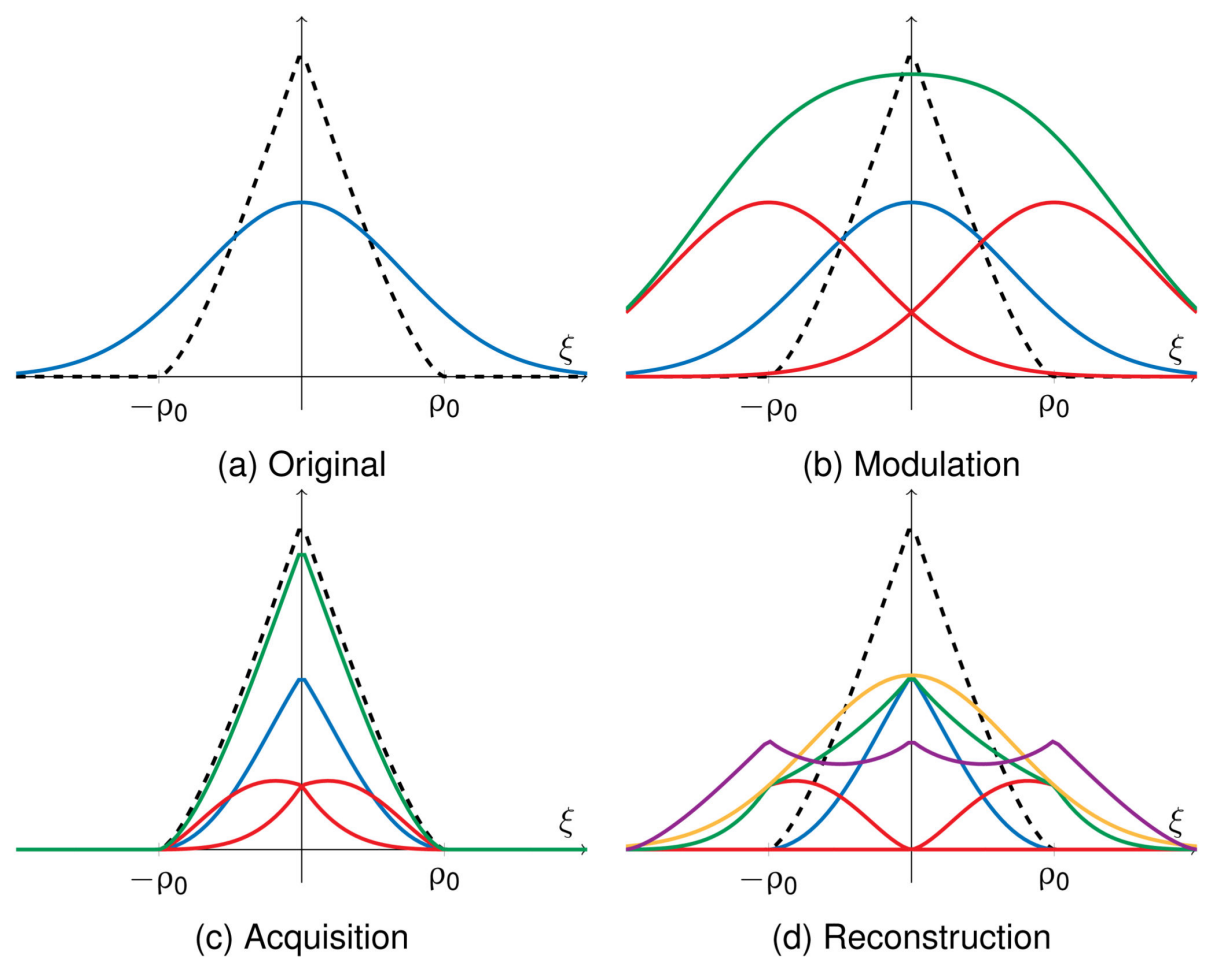
Principle of structured illumination microscopy illustrated in one dimension. (a) Spectrum of *x* in blue and the optical transfer function ${𝒜}_{0}$ in black (b) Spectrum of a modulated image **x**_*n*_ · (1 + cos(*ωn* + *φ*)) (green) as the sum of the three components 1, *e*^±*i*(*ωξ*+*φ*)^ (resp. blue and red) (c) Spectrum of the sum (green) and of the individual components (blue and green) after being filtered by the OTF of the optical system (d) Reconstruction of a superresolved image obtained by shifting the modulated components (red) and summing them (green). Finally, normalizing the components by taking into account the shape of the OTF (purple) allows us to recover the original image (yellow).

The convolution operator **A**_0_ models the point-spread function of the acquisition system, represented as a pseudo-circulant *N* × *N* matrix. In the sequel, we will use the notation **A** = **I**_*K*_ ⊗ **A**_0_ to represent the convolution of all modulated images. Moreover, when approximating the optical microscope by a perfect diffraction-limited 2D imaging system, we can model the optical transfer function (OTF) in widefield microscopy by the auto-correlation of the pupil function as [6, 23]:

(3) $$A_{0}\left(\varrho \right)=\{\begin{array}{ll} \frac{2}{\pi }\left(\arccos\left(\frac{\varrho }{2\varrho_{0}}\right)-\left(\frac{\varrho }{2\varrho_{0}}\right)\sqrt{1-{\left(\frac{\varrho }{2\varrho_{0}}\right)}^{2}}\right) & \varrho \leq \varrho_{0},\\ 0 & \text{otherwise}, \end{array},$$

where $\varrho =\sqrt{\xi_{1}^{2}+\xi_{2}^{2}}$ is the amplitude of the frequencies in polar coordinates and $\varrho_{0}$ the cutoff frequency. A profile of the OTF ${𝒜}_{0}(\varrho )$ is depicted in dashed black in Fig. 3. We can note that for any pair of signals whose spectrum only differs for frequencies greater than $\varrho_{0}$, both signals will be equal when viewed through the optical system. We therefore cannot assume the operator **A**_0_ to be injective.

The down-sampling operator **S**_0_ represented by a matrix of size *L* × *N*, where typically *L* = *N*/4, leads to down-sampling of a factor 2 in each dimension. Note that the images could be sampled at a higher rate at the acquisition time, however this would compromise the field of view and increase the noise level, since the number of photon per pixel would also decrease. In the rest of the text, down-sampling for the set of *K* images is represented by the operator **S** = **I**_*K*_ ⊗ **S**_0_.

To summarize our forward imaging model, we can now conveniently rewrite Eq. (7) as:

(4) $$\bar{\text{y}}=\text{SAM}\bar{\text{x}}.$$

where **ȳ** = [**ȳ**_1_, … , **ȳ**_*K*_]^T^ is the stack of noise-free images.

The principle of SIM imaging in the case of sinusoidal modulations is illustrated in Fig. 3. It depicts how the modulations amount to a shift in the Fourier domain (Fig. 3.b), that makes it possible for the optical system to capture information at frequencies above the cutoff frequency $\varrho_{0}$ (Fig. 3.c). By shifting back these components individually, a high resolution image is recovered. However, in order to obtain this highly resolved image (Fig. 3.d yellow curve) a normalization step equivalent to the ratio of the demodulated images (green curve) with the shifted OTFs (purple curve) is necessary, at the risk of amplifying the noise present in the acquired data.

Indeed, the acquired images are actually degraded by some random noise due to the photo–electron counting process (shot noise) and the thermal agitation of the electrons (dark current and readout noise). To take into account those degradations, a general noise model can be written as [24]:

(5) y=κp+n,

where *κ* is the overall gain of the acquisition system, **p** is a vector of Poisson distributed random variables of parameter (**ȳ** − *m*_DC_)/*κ* accounting for the shot noise and **n** a vector of Normally distributed random variables of mean *m*_DC_ and variance $\sigma_{\text{DC}}^{2}.$ The offset term *m_DC_* accounts for the baseline gray level that are characteristic of the sensor, while the variance $\sigma_{\text{DC}}^{2}$ of the additive white Gaussian noise summarizes several intensity-independent noise contributions such as dark current and readout noise. This formulation ensures that ${\text{lim}}_{N_{\ell \to \infty }}\frac{1}{N_{\ell }}\sum_{\ell =1}^{N_{\ell }}{\left(y\right)}_{\ell }\to \bar{y}$ for *N_ℓ_* different statistical realizations of the random vector (**y**)*_ℓ_*. The resulting distribution **y** is then the convolution of a Poisson distribution and a Normal distribution. Note that a direct use of a variance stabilization transform [25] would introduce nonlinearities, which would have a significant impact on the observation model (7) and make the reconstruction process intractable. The additive white Gaussian noise model cannot deal with variations of the noise level, especially considering that an image often has a high dymanic range (16bit), and a pure Poisson approximation does not account for the presence of additional readout noise. Therefore, we consider in Section 3.2, which are able to capture the specificities of joint Poisson-Gaussian noise.

## 3 Proposed approach

In this work, we focus on a general framework aiming to deal with possibly nonfinite data fidelity terms and nonsmooth regularization terms [26]. We formulate the estimation procedure as a minimization involving a sum of *Q* cost terms defined by:

(6) $$\hat{\text{x}}\in \underset{\text{x}\in C}{\text{Argmin}}\sum_{q=1}^{Q}f_{q}({\text{T}}_{q}\text{x}),$$

where *f_q_* are convex, closed and proper functions [27] from ℝ*^M_q_^* to ℝ ∪ {+∞}, *C* is a nonempty closed convex subset of ℝ^*N*^ (e.g. nonnegative solutions) and **T**_*q*_ operators represented as matrices of size *M_q_* × *N*. The cost terms *f_q_* (**T**_*q*_**x**) corresponding either to a data fidelity term or to a regularization term.

### 3.1 Primal–dual proximal minimization

When the involved functions are non-necessarily smooth, two main classes of algorithms can be derived to solve (6) and have been largely employed for solving inverse problems during the last decade: the alternating directions method of multipliers (ADMM) [28] or the Chambolle–Pock algorithm also known as primal–dual hybrid gradient (PDHG) [29–33]. Both strategies have in common to split the processing of the (**f**_*q*_)_1≤*q*≤*Q*_ and the (**T**_*q*_)_1≤*q*≤*Q*_ and to rely on the computation of the proximity operator [34] of each *f_q_*. We define the proximity operator prox_*f*_ : ℝ^*n*^ → ℝ^*n*^ of any closed proper convex function *f* : ℝ^*n*^ → ℝ ∪ {∞} as:

(7) $$\left(\forall \text{x}\in {\text{ℝ}}^{n}\right)\;{\text{prox}}_{f}\left(\text{x}\right)=\underset{\text{y}\in {\text{ℝ}}^{n}}{\text{arg}\;\text{min}}\left(\frac{1}{2}∥\text{x}-\text{y}{∥}_{2}^{2}+f\left(\text{y}\right)\right)$$

**Figure 4:**
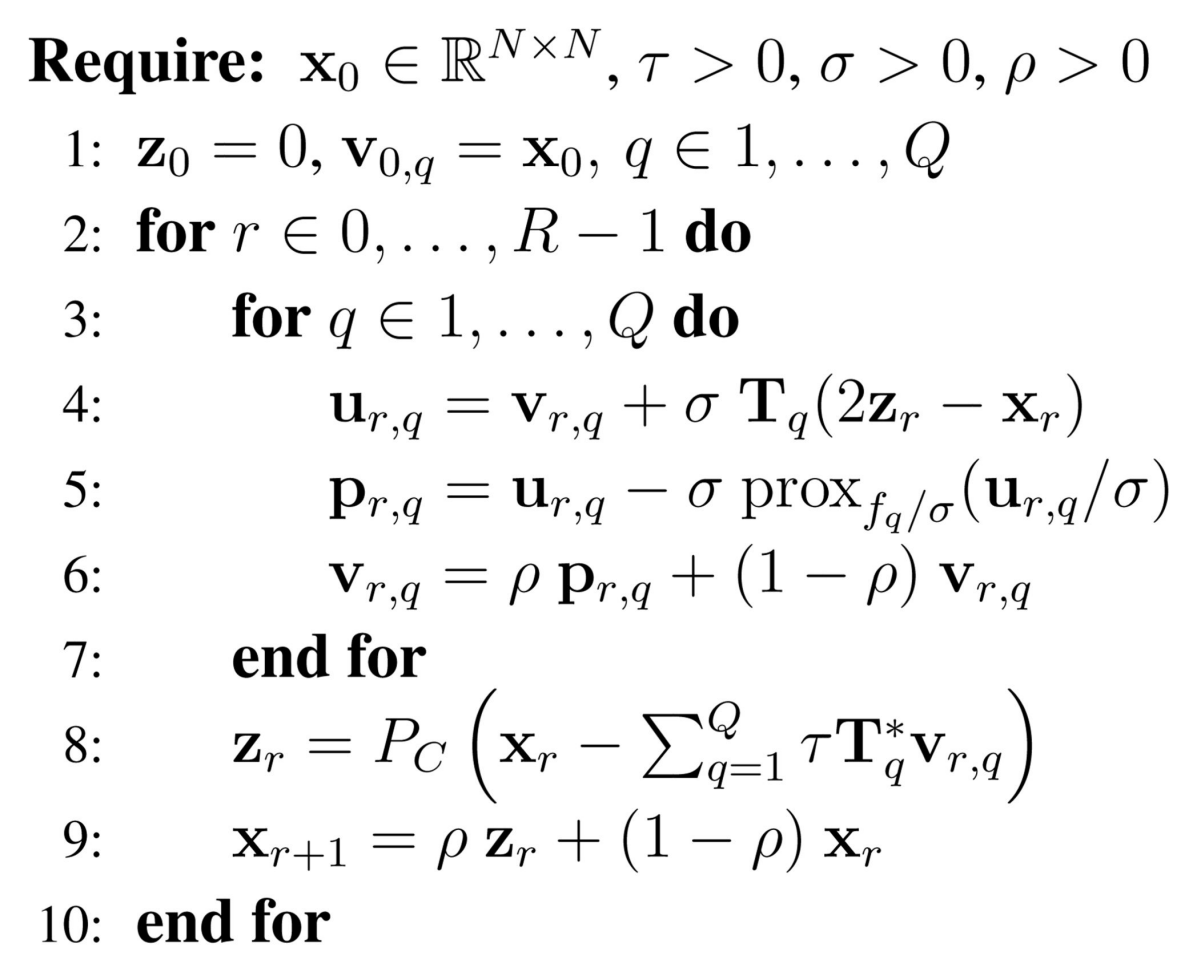
The primal–dual minimization algorithm proposed in [32] allows us to minimize the energy functional defined by Eq. (6) given that the proximity operator of the function *f_q_* and the operator **T**_*q*_ and its adjoint ${\text{T}}_{q}^{*}$ are defined. We can notice that this algorithm does not require the direct inversion of these operators.

Note that a large number of closed form expressions are known in the literature [27]. Some of them, useful for the study, are recalled in Appendix A. The major difference between both strategies comes from the way the operators (**T**_*q*_)_1≤*q*≤*Q*_ are handled. The ADMM requires to compute ${\left(\sum_{q=1}^{Q}{\text{T}}_{q}^{*}{\text{T}}_{q}\right)}^{-1}$ while the PDHG strategies avoid such a step. Note that since in general the operator associated to SIM imaging is not directly invertible,the ADMM would require an inner minimization procedure (*e.g.* the conjugate gradient) for the inversion of this operator. Finally, we can note that the PDHG can be formulated as a preconditioned ADMM [26]. Consequently, we solve Eq. (6) using the PDHG algorithm [29, 32, 33] (see Fig. 4) and consider several cases corresponding to the combination of function *f_q_* and operator **T**_*q*_. The algorithm has four parameters: the number of iteration *R*, *τ* ≈ *σ* and the acceleration *ρ* ∈ ]0, 2[. In practice, we use *R* = 500, *ρ* ≈ 2 and *σ* = 1/(*τ L*) where $L=\sqrt{\sum_{q=1}^{Q}∥T_{q}{∥}^{2}}$ [32]. The last parameter *τ* is set to 1 by default in our experiments but could be adapted for each objective function. Note that this parameter will only change the convergence rate.

In the next section, we will explicit the cost terms *f_q_* (**T**_*q*_**x**) corresponding to the proposed approach, while Appendix A details the other regularization terms tested in Section 4.2. We give the expression of the function *f_q_* and its associated proximity operator [34], and describe the operator **T**_*q*_ and its ajoints when needed. As a convention, we denote **z** = **T**_*q*_**x** the vector of length *M_q_* in the image space of **T**_*q*_. In practice, we will later consider only the combination of one data term along with one regularization term, while *C* will denote the nonnegativity constraint, which will be enforced directly on the iterates of the algorithm, see Step 8 in Fig. 4. Therefore, we can write the estimate as: **x̂** ∈ Argmin_**x**≥0_*f*_1_(**T**_1_**x**) + *f*_2_(**T**_2_**x**).

### 3.2 Poisson–Gaussian approximation

Handling Poisson–Gaussian noise is challenging as the resulting probability density function (p.d.f) is the convolution of the Poisson and Gaussian densities. Consequently, several strategies have been developed over the years to approximate the resulting p.d.f (See [35] for a recent review). The different approximations are more or less precise, depending on the relative amount of Gaussian and Poisson noise, and are more are less numerically tractable. Here, we propose to consider two approximations: the shifted Poisson model and the heteroscedastic Gaussian model approximation. One can notice a degree of symmetry between these two approaches, as the first one approximates Gaussian noise as Poisson noise with shifted intensity while the second one approximate Poisson noise by Gaussian noise with variance depending on the intensity of the signal.

#### Shifted Poisson model

Under a purely Poisson noise model assumption for the acquired data **y**, the negative log-likelihood would coincide up to a constant with the Kullback–Leibler (KL) divergence also called I-divergence [36] and can be expressed as $\text{z}={\left({\text{z}}_{n}\right)}_{1\leq n\leq \text{LK}}\mapsto f_{\text{Poisson}}\left(\text{z}\right)=\sum_{n=1}^{LK}f_{\text{Poisson}}^{\left(n\right)}\left({\text{z}}_{n}\right),$ where the component-wise function is defined by [37]:

(8) $$\left(\forall \gamma >0,\forall {\text{z}}_{n}\in \mathbb{R}\right)\;f_{\text{poisson}}^{\left(n\right)}\left({\text{z}}_{n}\right)=\{\begin{array}{ll} {\text{z}}_{n}-{\text{y}}_{n}{\text{log}\;\text{z}}_{n}, & {\text{z}}_{n},{\text{y}}_{n}>0,\\ {\text{z}}_{n}, & {\text{z}}_{n}>0\;\text{and}\;{\text{y}}_{n}=0,\\ \infty , & \text{otherwise}. \end{array}$$

In this configuration, the linear operator is **T**_Poisson_ = **SAM**. The proximity operator is given component-wise for *n* ∈ [1, *LK*] by:

(9) $$\left(\forall {\text{z}}_{n}\in \mathbb{R}\right)\;{\text{prox}}_{\gamma f_{\text{poisson}}^{\left(n\right)}}\left({\text{z}}_{n}\right)=\frac{1}{2}\left({\text{z}}_{n}-\gamma +\sqrt{{\left({\text{z}}_{n}-\gamma \right)}^{2}+4\gamma {\text{y}}_{n}}\right)$$

and the proximity operator ${\text{prox}}_{\gamma f_{\text{poisson}}}\left(\text{z}\right)={\left({\text{prox}}_{\gamma f_{\text{poisson}}^{\left(n\right)}}\left({\text{z}}_{n}\right)\right)}_{1\leq n\leq LK}$ is obtained by applying Eq. (9) to each component of the vector **z**.

In the case of Poisson–Gaussian noise, we can shift the Poisson likelihood as proposed by [38]. However, we would like to take into account the full model that we proposed in Eq. (5) with a Gaussian noise $\text{n}∼\text{N}(m_{\text{DC}},\sigma_{\text{DC}}^{2})$ and a gain *κ*. In order to do so, we seek a transformation of the form (**y** − *b*)/*a* with (*a*, *b*) ∈ ℝ^2^ so that the two first moments are matching after transformation $\text{𝔼}\left[\frac{\text{y}-b}{a}\right]=Var\left[\frac{\text{y}-b}{a}\right]$ in order to satisfy the intrinsic property of Poisson random variable. Choosing *a* = *κ* leads to $b=m_{\text{DC}}-\sigma_{\text{DC}}^{2}/\kappa$ and we can define then, the shifted Poisson data-fitting term as:

(10) $$(\forall {\text{z}}_{n}\in \mathbb{R})\;f_{\text{shifted-Poisson}}^{\left(n\right)}({\text{z}}_{n})=f_{\text{Poisson}}^{\left(n\right)}\left(\frac{{\text{z}}_{n}-b}{a}\right)$$

and the associated proximity operator component-wise for *n* ∈ [1, *LK*] as:

(11) $$(\forall {\text{z}}_{n}\in \mathbb{R})\;{\text{prox}}_{\gamma f_{\text{shifted}–\text{Poisson}}^{\left(n\right)}}({\text{z}}_{n})=a\;{\text{prox}}_{\gamma f_{\text{Poisson}}^{\left(n\right)}}\left(\frac{{\text{z}}_{n}-b}{a}\right)+b$$

using these two constants for the shifted Poisson approximation.

#### Heteroscedastic Gaussian noise model

As an approximation of the Poisson–Gaussian noise model, a weighted least-squares data term can be used to take into account the dependency between the variance of the noise level and the intensity of the signal. The weighted least-squares can be written as

(12) $$(\forall \text{z}\in {\mathbb{R}}^{LK})\;f_{\text{WLS}}(\text{z})=\frac{1}{2}{\left(\text{z}-\text{y}\right)}^{\text{T}}{\text{W}}^{-1}(\text{z}-\text{y}),$$

where **W** is a diagonal variance matrix of size *LK* × *LK* with elements (**w**_*n*_)_1≤*n*≤*LK*_ and the associated proximity operator is given by

(13) $$(\forall \gamma >0)(\forall \text{z}\in {\mathbb{R}}^{LK})\;{\text{prox}}_{\gamma f_{\text{WLS}}}(\text{z})={\left(\frac{{\text{w}}_{n}{\text{z}}_{n}+\gamma \;{\text{y}}_{n}}{{\text{w}}_{n}+\gamma }\right)}_{1\leq n\leq LK}.$$

Given the noise model defined by Eq. (5) the variance at each point *n* is given by:

(14) $$𝕍\text{ar}[{\text{y}}_{n}]=\kappa 𝔼[{\text{y}}_{n}]+\sigma_{\text{DC}}^{2}-\kappa \;m_{DC},$$

with $𝔼[y_{n}]$ and $\mathrm{𝕍ar}[y_{n}]$ the expectation and variance of the random variable **y**_*n*_. The weights are consequently given by:

(15) $${\text{w}}_{n}=\kappa \bar{\text{y}}+\sigma_{\text{DC}}^{2}-\kappa \;m_{DC}$$

where **ȳ** can be approximated by its noisy counterpart **y**.

#### Noise parameter estimation

If the CCD or sCMOS sensor have not been calibrated and the parameters *κ*, *m*_DC_ and *σ*_DC_ are unknown, we can follow the procedure described in [39] to estimate the required parameters. The variance of the noise $\mathrm{𝕍ar}[y_{n}]$ is estimated locally using a maximum of absolute deviation filter (MAD) computed on the pseudo-residuals (normalized Laplacian $\frac{1}{\sqrt{20}}\left({\text{D}}_{11}^{2}\text{y}+{\text{D}}_{22}^{2}\text{y}\right)$) of the image while the mean is estimated using a median filter. The linear regression allows us then to estimate the gain *κ* and the noise variance at the origin $e_{DC}=\sigma_{\text{DC}}^{2}-\kappa \;m_{DC}.$ Interestingly, these two parameters are sufficient to fully determine the noise model for both approximation.

#### Comparison of the associated likelihoods

**Figure 5:**
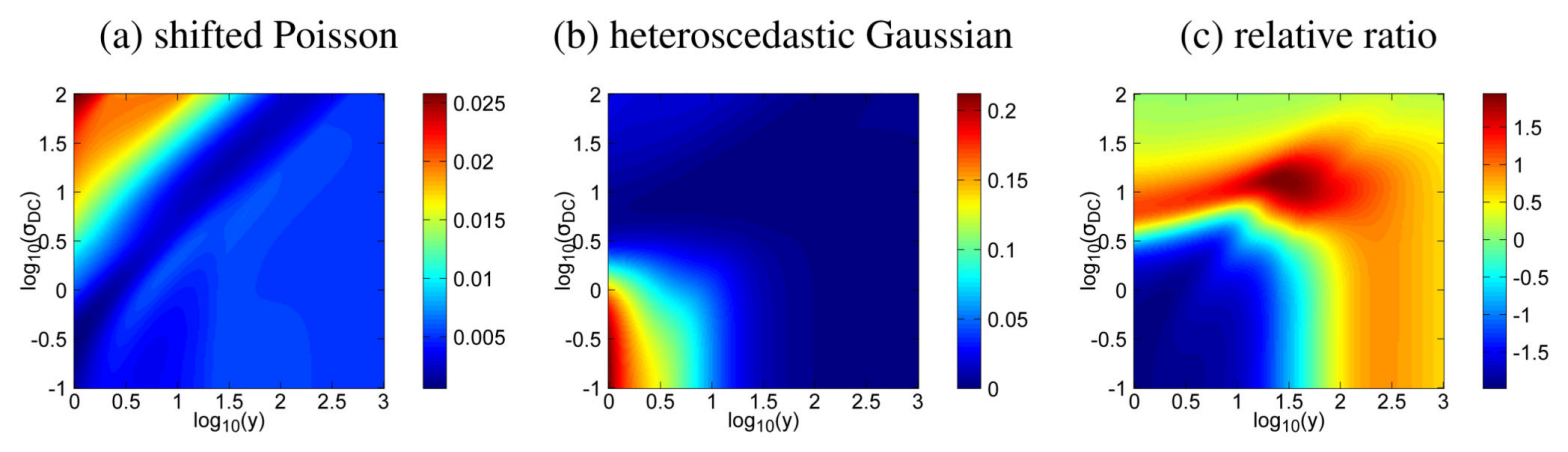
Poisson–Gaussian noise approximation error measured as the Hellinger distance between the exact likelihood and either shifted Poisson (a) or the heteroscedastic Gaussian (b) models. In (c) the relative ratio of the two errors highlights in blue the domain in the (**y**, *σ*_DC_) space where the shifted Poisson model outperforms the heteroscedastic approximation.

In order to gain some insight into these two approximations of the Poisson–Gaussian noise model, we can analyze the associated likelihoods. We present in Fig. 5 the Hellinger distance between the likelihoods of the exact model and the two approximations, in (a) and (b), as a function of the photo-electron count **ȳ** and readout noise *σ*_DC_ simplifying without loss of generality setting *κ* = 1 and *m*_DC_ = 0 for simplicity without loss of generality. The relative ratio shows that the likelihood of the shifted Poisson model is closer to the exact likelihood when the number of photons **ȳ** is approximately below 50 and the standard deviation of the readout noise is approximately below 5. Then a transition zone shown in red indicates that the heteroscedastic likelihood is closer to the exact model. Increasing further the photon count and the readout noise finally seems to stifle the difference between these two models as the relative difference tends to zero (lime green color). This analysis suggests that the approximation that should be used could be selected according to the regime where the data have been acquired.

### 3.3 Regularization by local dictionaries of patches

**Figure 6:**
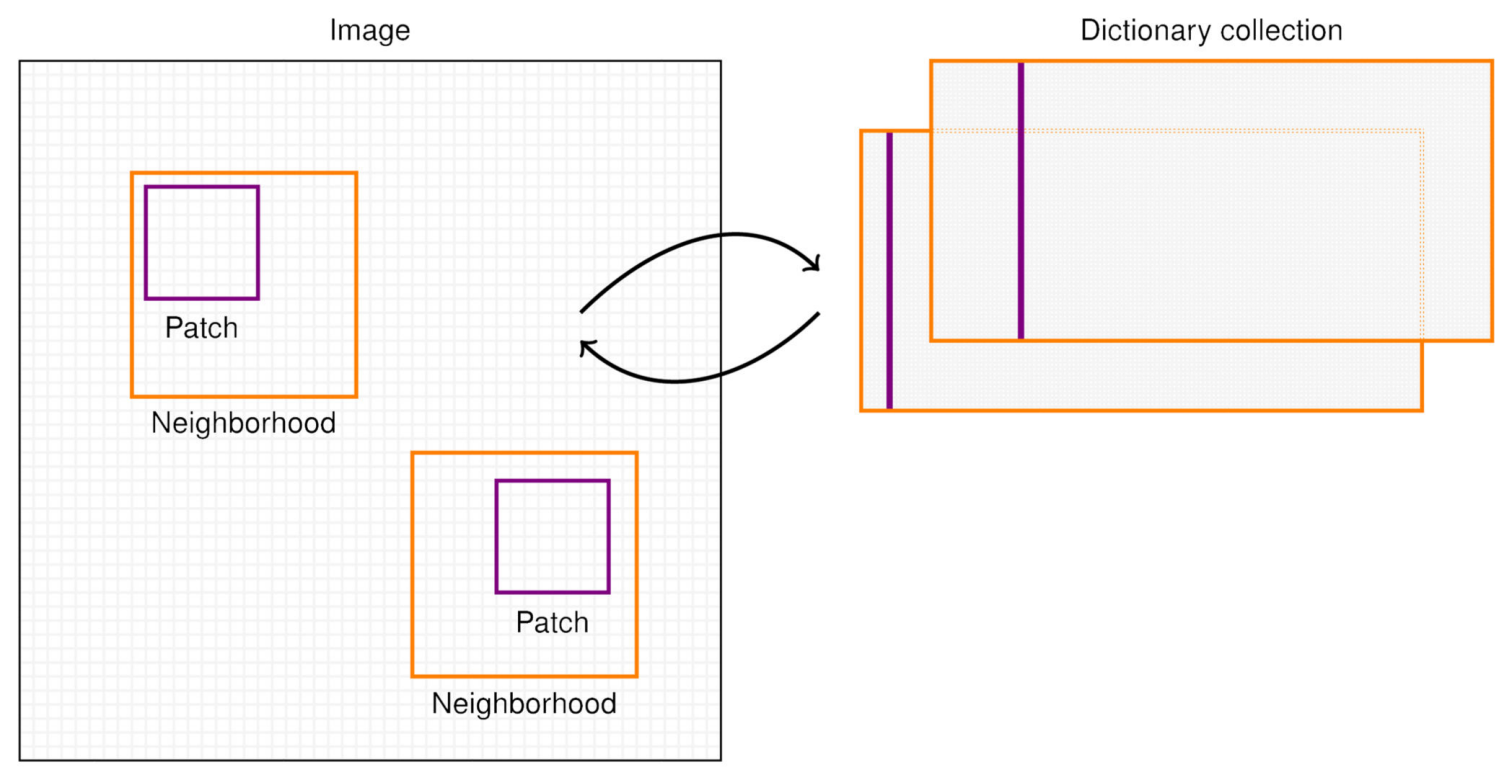
Illustration of the local dictionary of patch extraction. Neighborhood (16×16 pixels) depicted in orange are sampled every 8 pixels in each directions. For each neighborhood, each patches (violet) are collected and vectorized to form a matrix: the dictionary. Consequently, the image is represented as a collection of dictionaries.

We propose here to adapt the idea of online learning of sparse local dictionaries of patches in the context of inverse problem regularization by considering the nuclear norm of a patch extraction operator $T_{𝒫}$. This operator $T_{𝒫}$ maps all the *N_p_* × *N_p_* patches in each neighborhoods of dimension *N_w_* × *N_w_* into a matrices of dimension $N_{p}^{2}\times N_{w}^{2}.$ Dimension of $z=T_{𝒫}(x)$ can be then represented as a 4D array, where a $N_{p}^{2}\times N_{w}^{2}$ collection of patches corresponds to each 2D point of the image space. The adjoint of this operator is the projection of the overlapping collection patches onto the image. Note that the operator $T_{𝒫}$ does not depend on the content of **x** but is only parametrized by the windows and patch dimensions. As an illustration, let us consider the case of a 4 × 4 image and patches of size 2 × 2. Then the operator is:

$${\text{T}}_{P}\text{x}=\left[\begin{array}{cccc} x_{1} & x_{2} & x_{5} & x_{6}\\ x_{2} & x_{3} & x_{6} & x_{7}\\ x_{3} & x_{4} & x_{7} & x_{8}\\ x_{5} & x_{6} & x_{9} & x_{10}\\ x_{6} & x_{7} & x_{10} & x_{11}\\ x_{7} & x_{8} & x_{11} & x_{12}\\ x_{9} & x_{10} & x_{13} & x_{14}\\ x_{10} & x_{11} & x_{14} & x_{15}\\ x_{11} & x_{12} & x_{15} & x_{16} \end{array}\right]$$

and corresponds to the 9 possible translations of the patch of 4 elements, picking values within an image represented as a vector of 16 elements. The patch dictionaries are highly redundant and for computational efficiency, only a fraction of the possible neighborhoods can be considered by shifting the patch extraction window from its half in both directions. As an example, for a 512 × 512 images, the operator will map to a 128 × 128 field of 16 × 25 matrices corresponding to dictionaries of patches of size 4 × 4 extracted from neighborhoods of size 8 × 8. More generally, the total size of the collection of local dictionaries is given by $\frac{2n}{N_{w}}{\left(N_{w}-N_{p}+1\right)}^{2}N_{p}^{2}.$ In order to better illustrate this approach, the diagram shown in Fig. 6 represents the extraction of two dictionaries associated to the neighborhoods of two selected points in the image.

The nuclear norm ||**z**_*n*_||_*_ of **z**_*n*_ ∈ ℝ^2×2^ is defined as the *ℓ*_1_–norm of the diagonal matrix **Λ**_*n*_ such that ${\text{z}}_{n}={\text{U}}_{n}\Lambda_{n}{\text{V}}_{n}^{\text{T}}.$ Then, the associated proximity operator is given by [16]:

(16) $$(\forall {\text{z}}_{n}\in {\mathbb{R}}^{2\times 2})\;{\text{prox}}_{\gamma {\left\|\cdot \right\|}_{*}}({\text{z}}_{n})={\text{U}}_{n}{\text{prox}}_{\gamma {\left\|\cdot \right\|}_{1}}(\Lambda_{n}){\text{V}}_{n}^{\text{T}}.$$

The nuclear norm can be seen as a relaxed version of the case *ℓ*_0_-norm of the eigenvalues [40]. Note that the Schatten norm ${𝒮}_{p}$ of the Hessian operator with *p* = 1 described in Appendix A and introduced in [13] is identical to the nuclear norm.

## 4 Results

### 4.1 Influence of the patch and neighborhood size

We proposed here to evaluate the influence of the patch and neighborhood size of the proposed patch-based regularization approach. Table 1 gives the dictionary size factor, PSNR and elapsed time for 3 combinations of patch and neighborhood size. We can observe that the two first are equivalent in term of computation time, with the second one providing a slightly better PSNR. Using a combination of larger patch and neighborhood increases by a factor 3 the computation time while not improving the PSNR. Finally, we can note the dependency of the optimal regularization parameter on the patch and neighborhood size. In the following, we will use a 8 × 8 patch size and a 16 × 16 neighborhood size.

### 4.2 Evaluation of data fitting term and regularization term

In order to evaluate the proposed reconstruction method, we consider alternative regularization approaches corresponding to different choices for the function *f*_2_ and the operator **T**_2_. The regularization cost functions are the following

- the squared *ℓ*_2_-norm of the gradient operator (*f*_2_( · ) = || · ||^2^, **T**_2_ = [**D**_1_, **D**_2_]^*T*^),
- the squared *ℓ*_2_-norm of the Laplacian operator $\left(f_{2}(\cdot )={\left||\cdot |\right|}^{2},{\text{T}}_{2}={\text{D}}_{11}^{2}+{\text{D}}_{22}^{2}\right)$ [6],
- the total variation as the *ℓ*_1_-norm of the gradient operator (*f*_2_( · ) = || · ||_1_, **T**_2_ = [**D**_1_, **D**_2_]^*T*^) [41, 42],
- the nonlocal total variation as the *ℓ*_1_-norm of the weighted nonlocal finite difference operator (*f*_2_( · ) = || · ||_1_, **T**_2_ = **T**_NL_) [14],
- the Schatten norm (with *p* = 1, that is the nuclear norm) of the Hessian operator with $\left(f_{2}(\cdot )={𝒮}_{1}(\cdot ),T_{2}=T_{ℋ}\right)$ [13].

The details of the associated proximity operators and the definition of the linear operators are given in Appendix A. Note that only the three first regularization terms have been previously tested in this context, while the NLTV and Schatten norm of the Hessian operator have not been applied to SIM image reconstruction. Although none of these regularization terms have been tested in the context of Poisson–Gaussian noise model for SIM image reconstruction, we can note that they have been considered in the context image deconvolution under a Poisson-Gaussian noise model but with a different algorithm [35].

**Figure 7:**
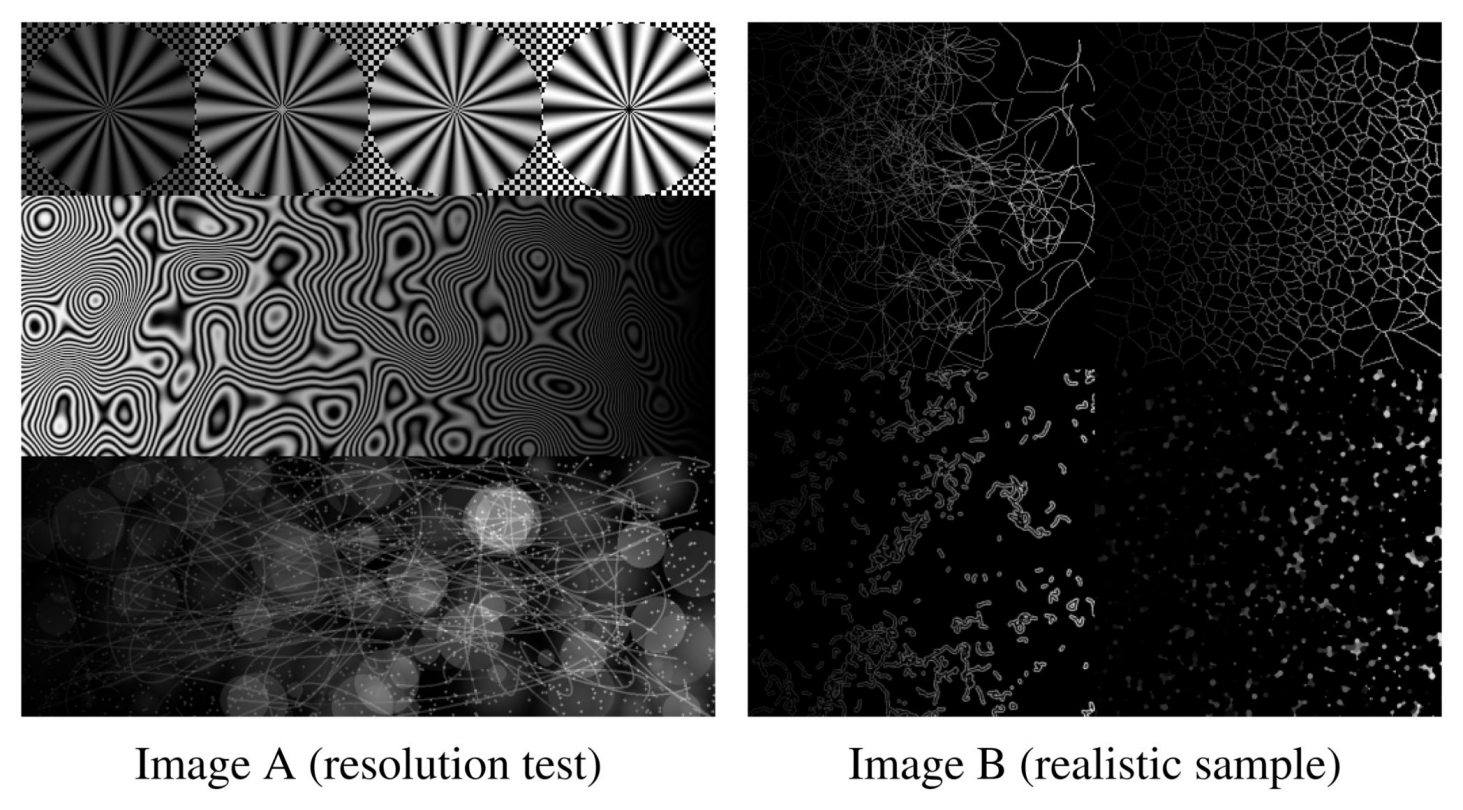
Test images used for evaluating the data-fitting and regularization terms.

We generated two test images (See Fig. 7). The first one aims at providing a visual assessment of the performance of the reconstruction in term of resolution, by integrating details at various frequencies. To check the data fitting under the Poisson–Gaussian noise assumption, we took care to integrate a large range of gray levels and to prevent any bias towards a particular regularization term, various realistic textures (points, blobs, lines, disks) are also used. The second image emulates several realistic intracellular features of interest for single cell imaging such as microtubules, endoplasmic reticulum, mitochondria and vesicles. The dynamic range of both images is [0, 255]. We simulated structured illumination images by using a down-sampling factor of 2 and a cut-off frequency *ρ*_0_ = 1.53 pixel^−1^. The modulations are composed of 3 equispaced phases and 3 equispaced angles with a frequency of 1 pixel^−1^. The images were finally corrupted by noise, as described in Eq. (5), with *κ* = 2, *m_DC_* = 0 and *σ_DC_* = 10. For each run, we used 500 iterations and we tested 20 values logarithmically spaced in the interval [0.01, 100] for the regularization parameters. We used the PSNR as a criterion in order to select the best image among the 20 results. All the implementation has been done using the MATLAB programming language.

To evaluate the performances of the reconstruction methods, we have considered the PSNR and the SSIM criteria. The PSNR being very sensitive to bias, we used a linear regression between the original image and the estimate to remove any systematic trend. More precisely the PSNR is defined as: PSNR(**x̄**, **x**) = 20 log_10_(255/||**x̄** − (**x** − *c*_0_)/*c*_1_)||^2^ where the coefficient *c*_0_ and *c*_1_ are estimated by by minimizing ||**x̂** − (*c*_0_ + *c*_1_**x**)||^2^.

**Figure 8:**
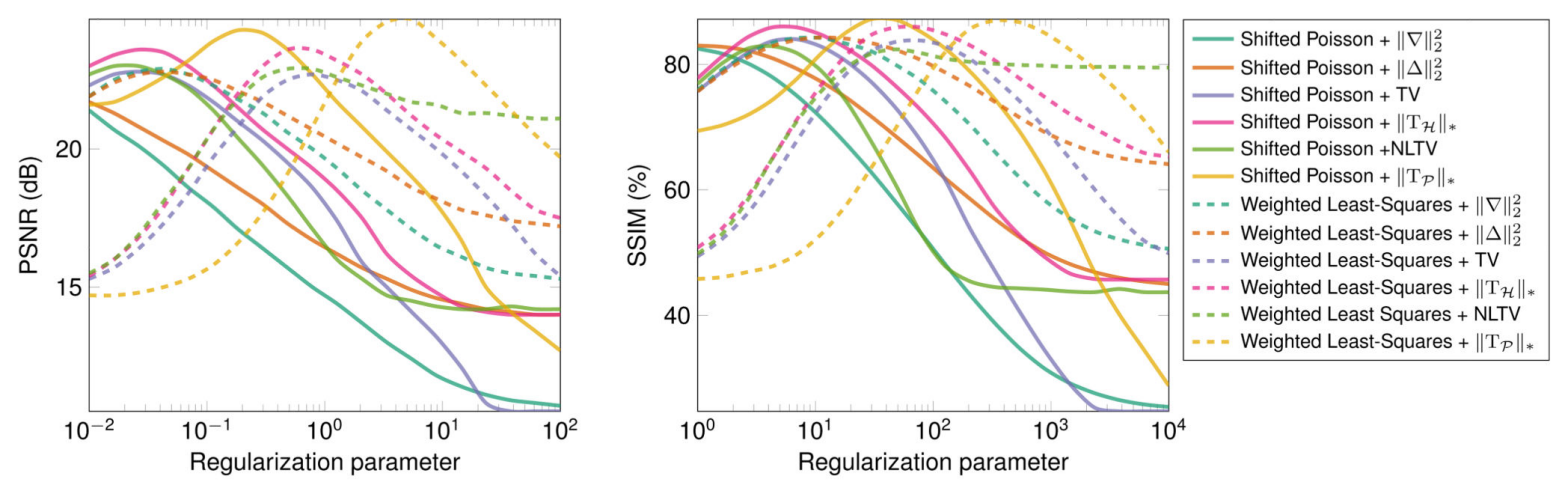
Evolution of the PSNR and the SSIM criterion as a function of the regularization parameters for the test image A.

Figure 8 displays the evolution of both criteria as a function of the regularization parameter. We can notice that if the difference between the two data-fitting terms are more apparent in term of PSNR than in term of SSIM while the optimal regularization parameter seems to be consistent between the two performance measures. Figure 4.2 shows the images corresponding to the best PSNR for the 12 cost functions for both test images with the PSNR values, the corresponding SSIM, the regularization parameter and the elapsed time. PSNR and SSIM values seem to correlate with the visual inspection of the images. In particular, high resolution information in the middle row of the image A seems to be better retrieved when using the proposed regularization approach. Note that the proposed regularization approach leads to significant computation times. On the one hand, all other approaches involve the optimized Matlab imfilter function, while on the other hand, the proposed approach requires many loops which are known to be slow in these condition. An implementation in another programming language using multi-threading would reduce the computational burden. Finally, best PSNR and SSIM values are displayed in Table 2 for each cost term and both images. We can see in bold that the best data fitting term would depend on the image and the performance measure while the proposed regularization consistently outperforms the other ones. Note that a comparison with recent methods taking advantage of the flexibility of the Bayesian framework for the formulation of the inverse problem [43] would be well suited for the reconstruction of blocky object, with sharp and sudden changes of intensity [44].

### 4.3 Modulation pattern

**Figure 10:**
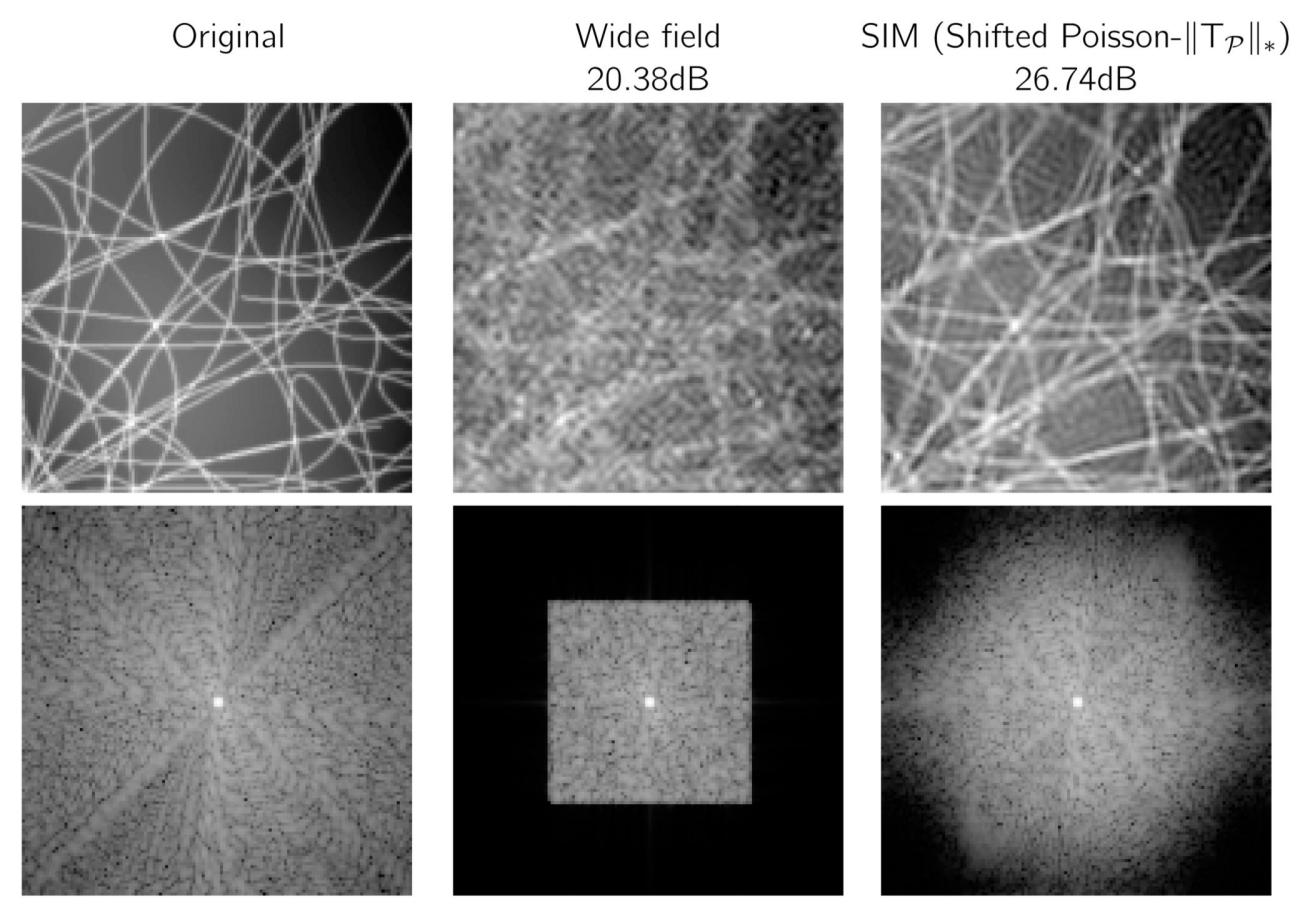
Reconstruction of a simulated 3 SIM images with a reduced number of images using only 3 modulation orientations and no phase shift. The second row displays the corresponding power spectra.

As described in [6], one advantage of considering the SIM image reconstruction as an inverse problem lies in the ability to reduce the number of acquired images. As an example, we can consider a set of 3 images with different modulation orientations but no phase shift. This allows us to effectively reduce the imaging speed and photo-toxicity which are both limiting factors in fluorescence light microscopy. This is a nonideal case as the sum of the modulation is not a uniform image and that therefore the noise is not spatially uniform. Nonetheless the results displayed in Fig. 10 show that the sample is successfully recovered with PSNR of 26.75dB and that high frequency details are well estimated as shown on the power-spectrum on the second row.

### 4.4 Reconstruction of acquired data

We have tested the proposed approaches on acquired data. For this purpose, we used two commercial systems: the N–SIM from Nikon and the OMX from General Electrics. Both microscopes use a similar approach for performing SIM imaging and rely on the use of a diffraction grating which is optically conjugated with the object plane.

**Figure 11:**
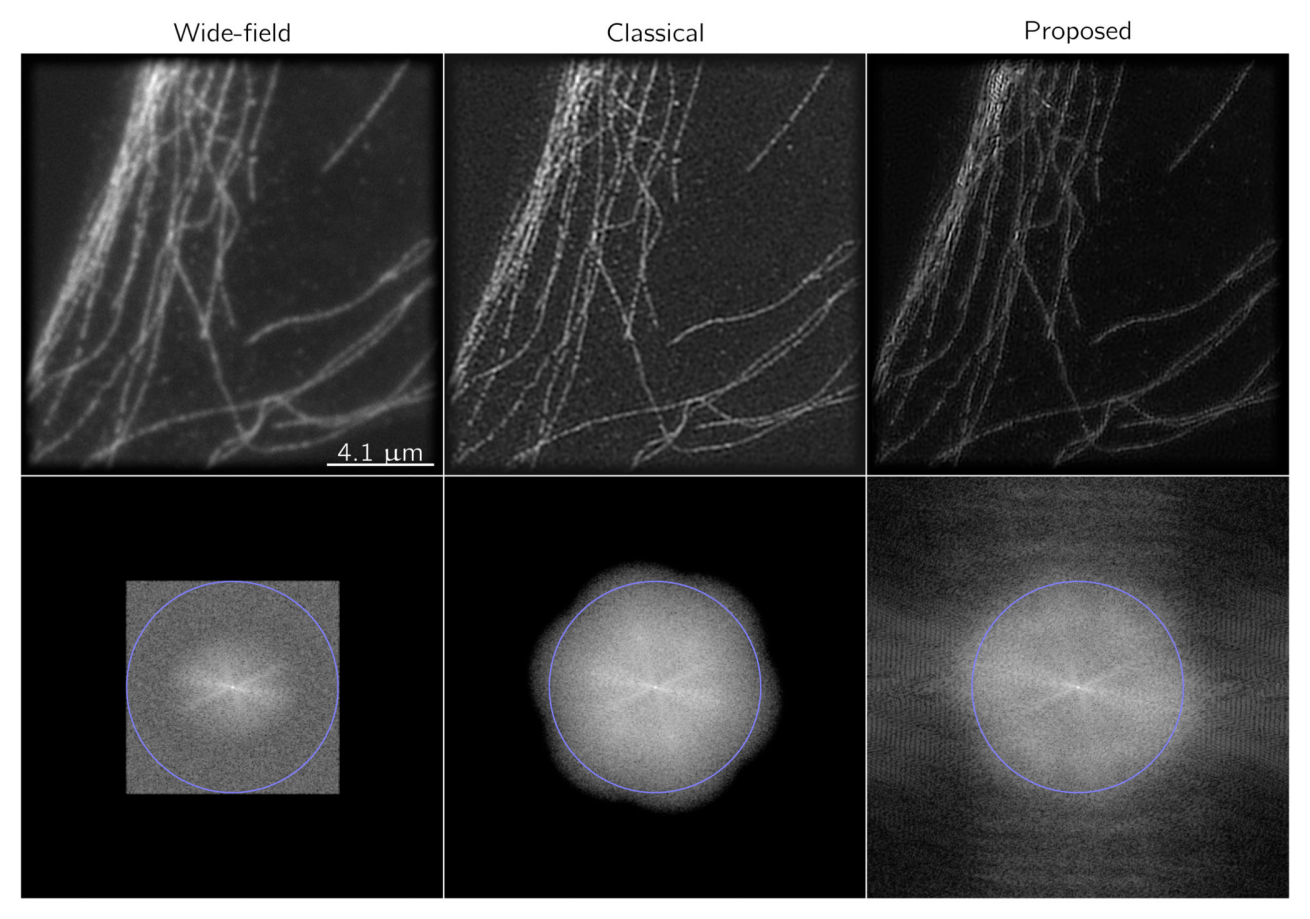
Reconstruction of acquired fluorescently labeled tubulin cell with a NSIM microscope. Structured illumination microscopy allows us to reveal the crossing of fibers with more details than the widefield image. The proposed approach is able to handle the noise and reduce the artifacts observed in the linear reconstruction (here the weighted least-square data fitting term was used). On the second row, the power spectrum is displayed as reveals the increased support in the frequency domain. The blue circle correspond to the resolution 128 μm.

The N–SIM is equipped with a 100× (1.49 N.A.) objective and a 2.5× lense is set on the camera port. A Xion Ultra 897 EMCCD camera from Andor Technology Ltd was on the detection path leading to a pixel-size of ~ 64 nm in the final image. A FluoCell prepared slide #2 with BPAE cells with Mouse Anti-*α*-tubulin was imaged and the results obtained with the linear and the convex nonsmooth reconstruction with a Poisson data term and a *local patch dictionary* regularization are shown in Fig. 11 along, with the “wide–field” image obtained by averaging the nine acquired images. On this image, we can notice that filaments appear much thinner on the nonsmooth estimate than on the linear one. We can also observe that the power spectrum seems to have a larger support.

The OMX microscope is equipped with a 100× (1.4 N.A.) objective coupled with a 2× lense on the camera port. This time a Evolve 512 from Photometrics was used and the final pixel-size in the image is ~ 80 nm. A FluoCell prepared slide #1 with BPAEC cells with F-actin stained with Alexa fluor 488 phalloidin. Once again, both linear and the proposed nonsmooth convex reconstruction methods reveal an increased resolution. Varying the regularization parameter for the linear method does not allow to reduce noise without inducing a loss of resolution. The proposed method allows us to achieve a much better compromise in this respect and clearly outperforms the linear approach.

## 5 Conclusion

We have proposed a new method for structured illumination image reconstruction. We have considered a primal–dual algorithm, which does not require the direct inversion of the forward operator, as this one is too large to be directly handled. In this framework, we have proposed a new regularization based on learning local patch dictionaries. Two valid approximations of the Poisson–Gaussian noise were tested and combined with several regularization to evaluate the performance of the proposed approach. The results show that the proposed approach leads to a significant improvement in terms of PSNR. Being able to better handle the noise perturbation make it possible to increase the resolution and the sensitivity of SIM images. We did not address the problem of the modulation parameter estimation, which can impact the quality of the reconstructed images [45]; we leave this study for future work. Finally, we have seen that the computation time associated to the proposed regularization are high. However, its implementation could be easily parallelized taking advantage of the multi-core architecture of modern CPUs and GPUs.

## Supplementary Material

Appendix

## Figures and Tables

**Figure 9:**
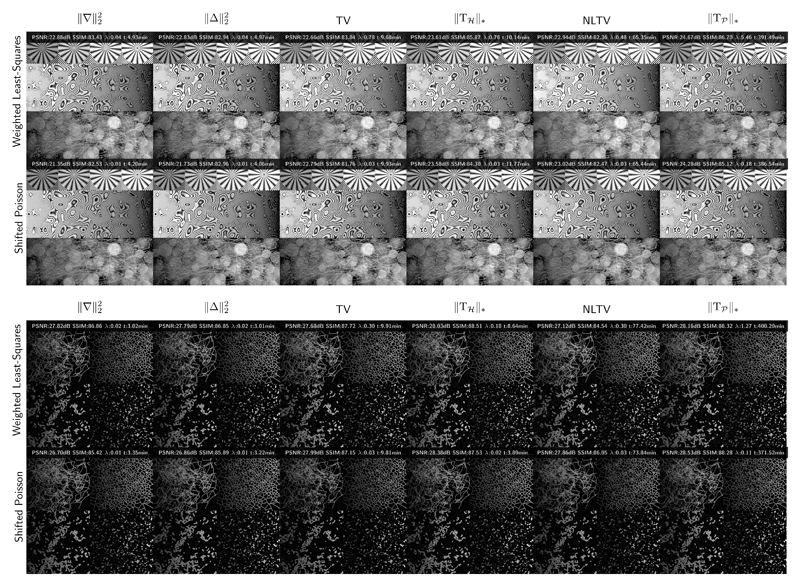
Reconstruction of the two test images with PSNR, SSIM, regularization parameter and elapsed time for the best PSNR. Images are displayed with gamma correction of 0.5 in order to better appreciate the details in the low end of the dynamic range.

**Table 1:** Influence of patch and neighborhood size. The dictionary size factor is a multiple of the initial image size.

Patch	Neighborhood	Dictionary	*λ*	PSNR (dB)	Elapsed time (s)
4 × 4	8 × 8	100	1.0	14.25	105
8 × 8	16 × 16	648	2.0	14.34	106
16 × 16	32 × 32	4624	2.0	14.11	375

**Table 2:** Best PSNR (dB) / SSIM (%) performance for both test images.

	$$\|\nabla {\|}_{2}^{2}$$	$$\|\Delta {\|}_{2}^{2}$$	TV	‖T*_ℋ_*‖_*_	NLTV	${{\|T}_{𝒫}\|}_{*}$
Shifted Poisson	21.35 / 82.53	21.73 / 82.96	22.79 / 84.00	23.58 / 85.94	23.02 / 82.95	24.28 / **87.18**
Weighted Least-Squares	22.88 / 84.20	22.83 / 84.30	22.66 / 83.84	23.61 / 85.94	22.94 / 82.36	**24.67** / 87.00

	$$\|\nabla {\|}_{2}^{2}$$	$$\|\Delta {\|}_{2}^{2}$$	TV	‖T*_ℋ_*‖_*_	NLTV	${{\|T}_{𝒫}\|}_{*}$
Shifted Poisson	26.70 / 85.42	26.86 / 85.89	27.99 / 87.59	28.38 / 88.08	27.86 / 86.52	**28.53** / 88.42
Weighted Least-Squares	27.82 / 86.86	27.79 / 86.97	27.68 / 87.81	28.03 / 88.51	27.12 / 85.57	28.16 / **88.61**
