# Supplementary material for: Nonsmooth Convex Optimization for Structured Illumination Microscopy Image Reconstruction
Source: Inverse Probl. Author manuscript; Available in PMC 2018 Sep 1. (PMC6075701; doi:10.1088/1361-6420/aaccca)
Supplement: Appendix [file NIHMS78519-supplement-Appendix.pdf]

Accepted Manuscript

Appendix A. Cost terms

We list here the implementation details for the other tested cost functions used in the numerical experiments.

Least-squares SIM (LS) When considering an additive Gaussian white noise model, the negative log-likelihood leads to a least-squares approach. The least-squares data term for SIM imaging is defined by $\frac{1}{2}\|\mathbf{y}-\mathbf{SAMx}\|_2^2$, corresponding to the combination of the function

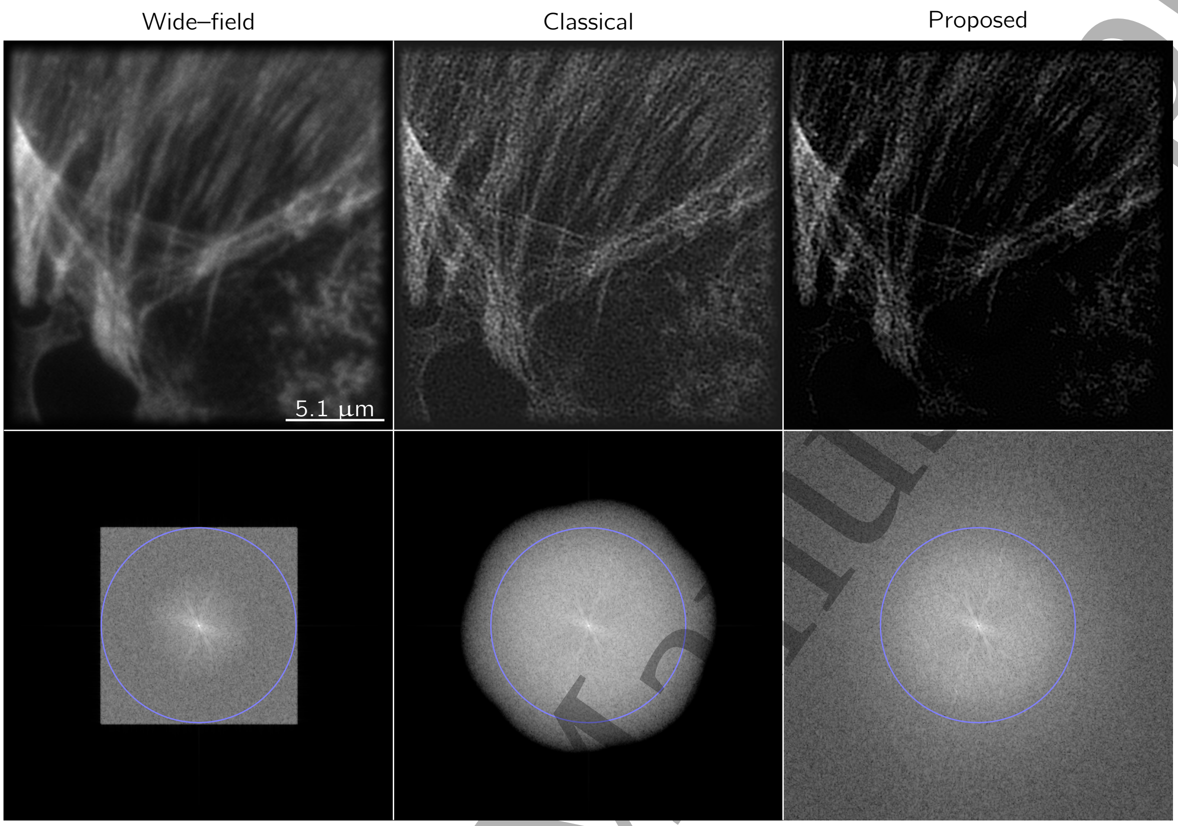

Figure 12. Reconstruction of acquired F-actin fluorescently labeled cell with the OMX setup. The fine and dense network structure of the actin cytosqueleton is better resolved when using the proposed approach. Corresponding power spectra are displayed in the second row.

$f_{\mathrm{LS}} = \frac{1}{2}\|\cdot - \mathbf{y}\|_2^2$ and the linear operator $\mathbf{T}_{\mathrm{LS}} = \mathbf{SAM}$. The proximity operator [34] associated to f_{LS} is then [27] : $\forall \gamma > 0, \forall \mathbf{z} \in \mathbb{R}^{LK}, \mathrm{prox}_{\gamma f_{\mathrm{LS}}}(\mathbf{z}) = (\mathbf{z} + \gamma\, \mathbf{y})/(1+\gamma)$.

Gradient squared ℓ_2–norm ($\|\nabla\|^2$) While more efficient algorithms exist for minimizing the squared ℓ_2-norm of the gradient of $\mathbf{x}$, especially combined with a least-squares data term, we may still use the proposed approach. In this case, the operator is defined by the two first order derivatives along the horizontal $\mathbf{D}_1$ and vertical $\mathbf{D}_2$ directions, stacked together as $\mathbf{T}_{\mathcal{D}} = [\mathbf{D}_1, \mathbf{D}_2]^{\mathrm{T}}$. The adjoint of this operator is then the opposite of the divergence operator defined as $\mathbf{T}_{\mathcal{D}}^* = \mathbf{D}_1^{\mathrm{T}}\mathbf{z}_1 + \mathbf{D}_2^{\mathrm{T}}\mathbf{z}_2$ where $\mathbf{z}_1$ and $\mathbf{z}_2$ are the gradient components. The gradient $\mathbf{D}_1$ and are $\mathbf{D}_2$ computed using a forward finite differences scheme and their adjoints $\mathbf{D}_1^{\mathrm{T}}$ and $\mathbf{D}_2^{\mathrm{T}}$ are backward finite differences with Neumann boundary conditions in both cases. For Tikhonov regularization, the associated function is then the squared ℓ_2–norm, i.e. $f_{\mathcal{D}} = \|\cdot\|^2$ whose proximity operator in this case is given by $\mathrm{prox}_{\gamma f_{\mathcal{D}}}(\mathbf{z}) = \mathbf{z}/(1+\gamma)$ for every $\mathbf{z} \in \mathbb{R}^{2N}$.

Laplacian squared ℓ_2–norm ($\|\Delta\|^2$) A Laplacian squared ℓ_2–norm regularization was introduced in [6] for SIM image reconstruction. We can consider this regularization using the proposed minimization algorithm by combining the squared ℓ_2–norm with the Laplacian operator $\mathbf{T}_{\mathcal{L}} = \mathbf{D}_{11}^2 + \mathbf{D}_{22}^2$ where $\mathbf{D}_{11}^2$ and $\mathbf{D}_{22}^2$ are the second order derivatives

Accepted Manuscript

Table 1. Detailed notations used in the paper.

n	index of the component of a vector (e.g. $\mathbf{x}$)	$\mathbf{I}_K$	$K \times K$ identity matrix
k	index for modulations	$\mathbf{M}_k$	modulations (diagonal matrix)
q	index of cost term (integer)	$\mathbf{A}_0$	point spread function (matrix)
r	iteration counter of an algorithm	$\mathbf{S}_0$	down-sampling (matrix)
N	pixel number of $\mathbf{x}$	$\mathbf{y}$	measurements (stacked vector)
L	pixel number of $\mathbf{y}$	$\mathbf{M}$	stacked modulations
K	number of modulations	$\mathbf{A}$	stacked point spread function
Q	number of cost term	$\mathbf{S}$	down-sampling (stacked matrix)
R	number of iterations	$\mathbf{W}$	diagonal weight matrix
T	number of translations (NLTV)	$\mathbf{p}$	number of photo electrons (stacked vector)
ϱ	radial frequency	$\mathbf{n}$	Gaussian white noise (stacked vector)
ξ_1, ξ_2	frequencies	$\mathbf{\Phi}_k$	phase matrix
κ	camera gain	$\mathbf{\Phi}$	stacked phase matrix
μ_{DC}	dark current noise mean offset	$\mathbf{\Omega}$	stacked frequency matrix
σ^2_{DC}	dark current noise variance	$\mathbf{L}$	Operator for linear regularized least-squares
ω_k	pulsation $2\pi\xi$ of the modulation	$\mathbf{D}_1$	finite difference operator
φ_k	phases of the modulation	$\mathbf{D}^2_{11}$	second order finite difference operator
τ, σ, ρ	algorithm parameters	$\mathbf{T}_q$	generic operator in the cost term
$\bar{\mathbf{x}}$	ground truth image $\mathbf{x}$	$\mathbf{T}_{\mathcal{D}}$	Stacked gradient operator
$\mathbf{x}$	estimated image (vector)	$\mathbf{T}_{\mathcal{L}}$	Laplacian operator
$\mathbf{y}_k$	measurements (vector)	$\mathbf{T}_{\mathcal{H}}$	Stacked Hessian operator
$\mathbf{z}$	the image of $\mathrm{T}_q\mathbf{x}$ (vector)	$\mathbf{T}_{\mathcal{P}}$	Patch extraction operator
$\mathbf{I}_K$	identity matrix	f_q	function in the cost term

in the horizontal and vertical directions. Note that the Laplacian operator is self-adjoint. Furthermore, we can use here the same function $f_{\mathcal{L}} = \|\cdot\|^2$ and the associated proximity operator as for Tikhonov regularization. Note that in the context of this study, unlike in [6], we do not consider the posterior mean estimate but only a maximum *a posteriori* (MAP) estimate.

Total variation (TV) The total variation seminorm can be defined as the ℓ_1–norm of the gradients of $\mathbf{x}$ [41,42]. Therefore, we can use this time the same operator $\mathbf{T}_{\mathcal{D}}$ as for Tikhonov regularization, but with a different function f. Indeed, in order to achieve an isotropic total variation, a vectorial form of the ℓ_1–norm denoted by $f_{\mathrm{TV}} = \|\cdot\|_{1,2}$ should be applied, by considering the two gradient components as a vector [27]:

$$(\forall \mathbf{z} = [\mathbf{z}_1^\top, \mathbf{z}_2^\top]^\top) \in \mathbb{R}^{2N} \quad \|\mathbf{z}\|_{1,2} = \textstyle\sum_{n=1}^{N} \sqrt{[\mathbf{z}_1]_n^2 + [\mathbf{z}_2]_n^2}. \qquad \text{(A.1)}$$

Then the proximity operator is applied component-wise for $n \in [1, N]$ as:

$$(\forall \mathbf{z}_n \in \mathbb{R}^2) \quad \mathrm{prox}_{\|\cdot\|_{1,2}}(\mathbf{z}_n) = \begin{cases} \mathbf{z}_n - \frac{\gamma \mathbf{z}_n}{\sqrt{[\mathbf{z}_1]_n^2 + [\mathbf{z}_2]_n^2}}, & \sqrt{[\mathbf{z}_1]_n^2 + [\mathbf{z}_2]_n^2} \geq \gamma \\ 0 & \text{otherwise.} \end{cases} \qquad \text{(A.2)}$$

Accepted Manuscript

Schatten norm of the Hessian operator ($\mathcal{S}_p(\mathrm{T}_{\mathcal{H}})$) Recently, a new regularization based on the Schatten norm of the Hessian operator has been proposed [13]. This approach has been developed in order to reduce the staircase artifacts observed with total variation regularization.

In order to include this regularization constraint, we consider the Hessian operator defined at each location $n \in \{1, \dots, N\}$ as:

$$[\mathbf{T}_{\mathcal{H}}\mathbf{x}]_n = \begin{bmatrix} [\mathbf{D}_{11}^2\mathbf{x}]_n & [\mathbf{D}_{12}^2\mathbf{x}]_n \\ [\mathbf{D}_{12}^2\mathbf{x}]_n & [\mathbf{D}_{22}^2\mathbf{x}]_n \end{bmatrix} \tag{A.3}$$

and composed of the second order derivative along horizontal, diagonal and vertical direction denoted respectively $\mathbf{D}_{11}^2$, $\mathbf{D}_{12}^2$ and $\mathbf{D}_{22}^2$. The adjoint of this operator is defined by:

$$\mathbf{T}_{\mathcal{H}}^*\mathbf{z} = \mathbf{D}_{11}^{2*}\mathbf{z}_{11} + \mathbf{D}_{12}^{2*}(\mathbf{z}_{12} + \mathbf{z}_{21}) + \mathbf{D}_{22}^{2*}\mathbf{z}_{22} \tag{A.4}$$

for every

$$\mathbf{z} = \begin{bmatrix} \mathbf{z}_{11} & \mathbf{z}_{12} \\ \mathbf{z}_{21} & \mathbf{z}_{22} \end{bmatrix}$$

where $\mathbf{z}_{11}$, $\mathbf{z}_{12} = \mathbf{z}_{21}$ and $\mathbf{z}_{22}$ represent the four components of the Hessian operator, each of size $\mathbb{R}^N$.

In a similar way to the nuclear norm, the Schatten norm $\mathcal{S}_p$ of $\mathbf{z}_n \in \mathbb{R}^{2\times 2}$ is defined as the ℓ_p–norm of the diagonal matrix $\mathbf{\Lambda}_n$ such that $\mathbf{z}_n = \mathbf{U}_n\mathbf{\Lambda}_n\mathbf{V}_n^{\mathrm{T}}$, and the proximity operator has the following expression [16]:

$$(\forall \mathbf{z}_n \in \mathbb{R}^{2\times 2}) \quad \mathrm{prox}_{\gamma\mathcal{S}_p}(\mathbf{z}_n) = \mathbf{U}_n \mathrm{prox}_{\gamma\|\cdot\|_p}(\mathbf{\Lambda}_n)\mathbf{V}_n^{\mathrm{T}}. \tag{A.5}$$

Nonlocal total variation (NLTV) The nonlocal total variation (NLTV) penalization was introduced in [14] and extended to various inverse problems in [15, 46] by considering differential operators defined on the graph associated to the sites of the image grid. It was also recently extended to multispectral images in [16]. The operator associated to the NLTV regularization can be described as weighted nonlocal gradients defined as [47]:

$$[\mathbf{T}_{\mathrm{NL}}\mathbf{x}]_n = \begin{bmatrix} [\mathbf{W}_1(\mathbf{F}_1\mathbf{x} - \mathbf{x})]_n \\ \vdots \\ [\mathbf{W}_T(\mathbf{F}_T\mathbf{x} - \mathbf{x})]_n \end{bmatrix} \tag{A.6}$$

where for $t \in 1, \dots, T$, we define some diagonal weight matrices as functions of the distance between patches $\mathbf{W}_t = \mathrm{diag}\left(\exp\left(-\frac{1}{\eta}\mathbf{B}(\mathbf{F}_t\tilde{\mathbf{x}} - \tilde{\mathbf{x}})^2\right)\right)$ with $\mathbf{F}_t$ a translation operator and $\mathbf{B}$ a convolution by a lowpass filter such as a box-filter, or a Gaussian filter and η a positive scalar. The image $\tilde{\mathbf{x}}$ can be obtained by minimizing the classical total variation for example. Note that the computation of the convolution could be done using separable recursive filters as proposed in [48]. However, since the estimation of the weights is performed only once, this step is not critical in terms of computation time. The T translations $\mathbf{F}_t$ are chosen so that they describe a square neighborhood of size $N_w \times N_w$ while the operator $\mathbf{B}$ corresponding to an

Accepted Manuscript

image patch whose size $N_p \times N_p$ is given by the width of the support of the filter in the case of a box–filter. The adjoint of the operator $\mathbf{T}_{\mathrm{NL}}$ is defined by:

$$(\forall \mathbf{z} \in \mathbb{R}^{TN}) \quad \mathbf{T}_{\mathrm{NL}}^{*}\mathbf{z} = \sum_{t=1}^{T} \mathbf{W}_t(\mathbf{F}_t^{*} - \mathbf{I})\mathbf{z}_t, \tag{A.7}$$

where $\mathbf{F}_t^{*}$ with $t \in 1, \ldots, T$ are the translation with the corresponding opposite directions. The function associated to the NLTV regularization is a $\ell_{1,2}$–norm defined by:

$$(\forall \mathbf{z} \in \mathbb{R}^{TN}) \quad \|\mathbf{z}\|_{1,2} = \sum_{n=1}^{N} \left(\sum_{t=1}^{T} \mathbf{z}_{n,t}^{2} \right)^{\frac{1}{2}}. \tag{A.8}$$

The associated proximity operator is then defined by:

$$(\forall \mathbf{z}_n \in \mathbb{R}^{T}) \quad \mathrm{prox}_{\gamma\|\cdot\|_{1,2}}(\mathbf{z}_n) = \begin{cases} \mathrm{z}_n - \frac{\gamma \mathbf{z}_n}{\sqrt{\sum_{t=1}^{T} [\mathbf{z}_t]_n^2}}, & \sqrt{\sum_{t=1}^{T} [\mathbf{z}_t]_n^2} \geq \gamma \\ 0 & \text{otherwise.} \end{cases} \tag{A.9}$$

Accepted Manuscript
